# Models versus pathogens: how conserved is the FtsZ in bacteria?

**DOI:** 10.1042/BSR20221664

**Published:** 2023-02-10

**Authors:** Rachana Rao Battaje, Ravikant Piyush, Vidyadhar Pratap, Dulal Panda

**Affiliations:** 1 Department of Biosciences and Bioengineering, Indian Institute of Technology Bombay, Mumbai 400076, India; 2 National Institute of Pharmaceutical Education and Research, S.A.S. Nagar, Punjab 160062, India

**Keywords:** Anti-bacterial therapy, Anti-Microbial Resistance, Bacterial Cell division, FtsZ, Pathogenic bacteria

## Abstract

Combating anti-microbial resistance by developing alternative strategies is the need of the hour. Cell division, particularly FtsZ, is being extensively studied for its potential as an alternative target for anti-bacterial therapy. *Bacillus subtilis* and *Escherichia coli* are the two well-studied models for research on FtsZ, the leader protein of the cell division machinery. As representatives of gram-positive and gram-negative bacteria, respectively, these organisms have provided an extensive outlook into the process of cell division in rod-shaped bacteria. However, research on other shapes of bacteria, like cocci and ovococci, lags behind that of model rods. Even though most regions of FtsZ show sequence and structural conservation throughout bacteria, the differences in FtsZ functioning and interacting partners establish several different modes of division in different bacteria. In this review, we compare the features of FtsZ and cell division in the model rods *B. subtilis* and *E. coli* and the four pathogens: *Staphylococcus aureus, Streptococcus pneumoniae, Mycobacterium tuberculosis*, and *Pseudomonas aeruginosa*. Reviewing several recent articles on these pathogenic bacteria, we have highlighted the functioning of FtsZ, the unique roles of FtsZ-associated proteins, and the cell division processes in them. Further, we provide a detailed look at the anti-FtsZ compounds discovered and their target bacteria, emphasizing the need for elucidation of the anti-FtsZ mechanism of action in different bacteria. Current challenges and opportunities in the ongoing journey of identifying potent anti-FtsZ drugs have also been described.

## Introduction

Most bacteria divide by binary fission. One bacterial cell splitting into two daughter cells seems to be a simple process, but the research in this field spanning decades demonstrates the complexity of the process. Bacterial cell division involves a myriad of protein complexes that are spatiotemporally regulated in accordance with environmental, chemical, and intrinsic factors, all taking place in about 30 min!

**Figure 1:**
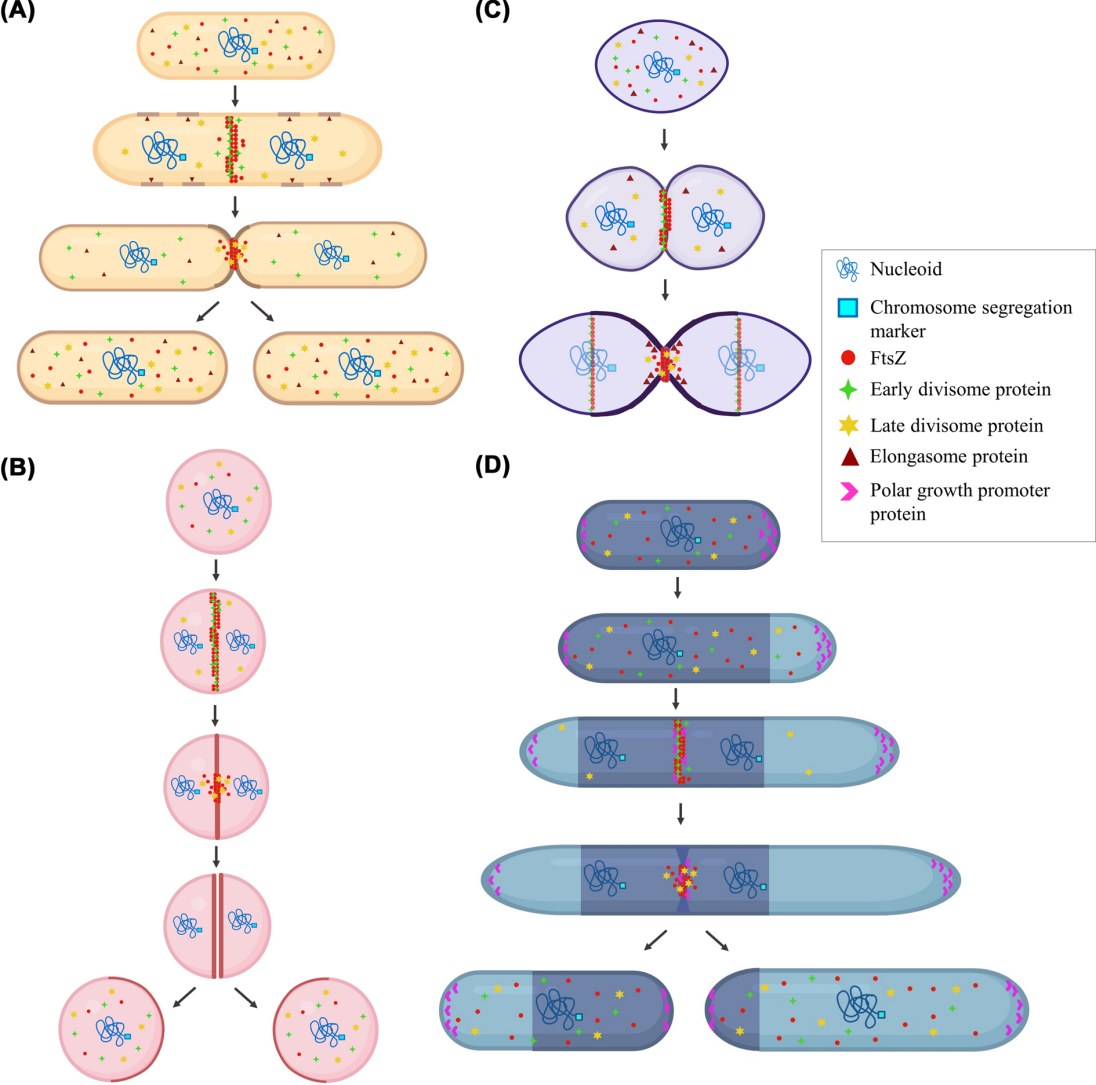
FtsZ as the central player in different modes of cell division (**A**) Division in model rods like *E. coli* and *B. subtilis* is carried out by the coordination of the elongasome machinery at different sites in the cell and by the divisome complex in the mid-cell region. On the direction of several mid-cell site markers, some associated with the nucleoid separation, FtsZ assembles into a central Z-ring in coordination with early divisome proteins to establish the divisome complex. The Z-ring coordinates septum formation and interacts with late divisome proteins to begin the septum peptidoglycan (PG) synthesis. Peripheral PG synthesis is carried out by the elongasome. Finally, membrane constriction leads to cytokinesis resulting in the formation of two daughter cells. (**B**) In the absence of elongasome machinery, cell division in cocci like *S. aureus* occurs through FtsZ-led division machinery only. Z-ring formation at the mid-cell site follows the septum formation in a centripetal pattern towards the centre of the cell. PG synthesis takes place in the septal region alone and final constriction leads to pinching off the two hemispherical lobes to give two daughter cells. The daughter cells immediately bulge out to form cocci each with one-half of the parent cell wall. (**C**) In ovococci like *S. pneumoniae*, the division is initiated by the assembly of initiation complex - FtsZ in a tight complex with early divisome proteins at the septal region. Cells have both elongasome machinery and divisome machinery present at the septal region, the coordination between the two leads to the formation of the septum and PG synthesis in the vicinity of the septum. The initiation complex assembles at the division site of the future daughter cells over the undivided nucleoid, thus giving rise to overlapping division cycles and cells mostly observed in diplococci state. (**D**) Asymmetrical cell division observed in *M. tuberculosis* is established by polar growth at one of the poles of the cell occurring at a faster rate than other. More Wag31, a protein that is associated with promoting polar growth, assembles at the old pole (established at previous division cycle) and coordinates accelerated growth (depicted in cyan). While growth at the new pole lags behind, the cell division process begins. Wag31 is also found to interact with FtsZ at the septum. The septum formation and membrane constriction proceed, and final ‘V-snapping’ results in the formation of two unequal daughter cells. The new poles are established after separation, while the other poles are now designated ‘old’ poles. (Images were created using BioRender.com)

A central protein that is responsible for orchestrating the cell division process is FtsZ, filamentous-temperature sensitive mutant Z. It is the bacterial homolog of the tubulin, the eukaryotic cytoskeletal protein [1]. When the bacterial cell is primed for division, FtsZ self-assembles into a dynamic structure at the mid-cell region called the Z-ring. Formed of several FtsZ polymers discontinuously bundled into treadmilling filaments, the Z-ring is responsible for the assembly of the divisome and the peptidoglycan (PG) synthesis complex at the site of cell division (Figure 1) [2–5].

The growing problem of anti-microbial resistance (AMR) has triggered the extensive search for novel antibiotics and alternative strategies for antibacterial therapy. Cell division in bacteria is an essential process presenting the central player- FtsZ, as an attractive alternative target for the design of antibiotics. Several anti-FtsZ compounds have been discovered and their mechanisms have been explored [1,6,7]. However, no FtsZ-targeting agent has been translated to the clinical stage. What are the scientists missing?

The rod-shaped bacteria, *Bacillus subtilis* and *Escherichia coli*, have been the model organisms for extensive studies on their cell shape and cell division processes. Most of the information on FtsZ’s role in cell division comes from these rods with widespread *in vivo* and *in vitro* studies [8–10]. Several recent reviews have covered the vast research in the field of FtsZ with extensive descriptions of FtsZ structure and function, and its potential as an antibiotic target [8–22]. The dynamics of FtsZ and its regulation inside the cells is an ever-evolving field of research with new information added every day. But only a few of these are arising from FtsZ from other bacteria that include pathogenic species.

Even though FtsZ is a conserved protein throughout the bacterial species, there are several important differences even between *B. subtilis* and *E. coli* with respect to its regulation, interaction with other proteins, and FtsZ dynamics. These two organisms, although both rod-shaped, show dissimilarities within their FtsZ-mediated cell division process, demonstrating the need for understanding the cell division process at the species level. Considering the literature on bacterial pathogens and their cell shapes ranging from rods, and cocci, to ovoid, there is a significant gap in the knowledge of FtsZ and cell division in these organisms.

In this review, we highlight the differences in FtsZ characteristics between model bacteria, *E. coli* and *B. subtilis*, and some of the pathogenic bacteria, *Staphylococcus aureus, Streptococcus pneumoniae, Mycobacterium tuberculosis*, and *Pseudomonas aeruginosa*. We detail the known cell division and cell shape determinants of the bacteria in this review, focusing on FtsZ and its partners. We emphasize that a conserved protein like FtsZ, shows differential behavior and interactions in different bacteria leading us to ask the question—How ‘conserved’ is FtsZ? Our goal is to use the pathogenic bacteria as representatives to enumerate unique and dissimilar aspects of cell division to encourage the study of anti-FtsZ agents in the pathogenic bacteria. This would further lead to fast-tracking the development of alternative targets for anti-bacterial therapy and in time, bridge the gap between drug discovery and *in vivo* efficacy for clinical translation.

## What’s in a shape? The coordination of cell division in different bacteria

### Cell division in rod-shaped bacteria is coordinated by Elongasome-Divisome interplay

The model organisms, *B. subtilis* and *E. coli* are gram-positive and gram-negative rods, respectively, with an average division time of 20 min. A nosocomial pathogen *Pseudomonas aeruginosa* is an encapsulated gram-negative rod. It is a wound-related infectious agent also causing bacteremia, septic shock, and urinary tract infections. Recently, carbapenem-resistant *P. aeruginosa* has been listed for critical need for development of alternative strategies for treatment against it [23]. The presence of the lipopolysaccharide outer membrane in the bacteria allows it to restrict the entry of antibiotics, contributing to AMR development. Efflux pumps and antibiotic-inactivating enzymes are other factors [23]. *P. aeruginosa* being a rod exhibits a similar cell division behavior as *E. coli* and *B. subtilis*. Even though homologs of several proteins in *E. coli* are found in *P. aeruginosa*, their functional characterization lags far behind that of the model rods.

A typical rod, like *B. subtilis*, has two machineries for cell division and cell elongation (Figure 1A). The protein complex responsible for septation and division called the divisome, and the protein complex responsible for peripheral elongation that determines the shape of the bacteria, called the elongasome, coordinate with each other during the cell division cycle. Cell elongation complex comprises MreB, the primary protein responsible for recruiting proteins such as MreC, MreD, and penicillin-binding proteins (PBP) to various foci along the cell. MreB forms a patchy network of filaments that undergo circumferential motion and coordinates with the peptidoglycan synthases [24–26]. In *E. coli*, MreB and FtsZ have been reported to be involved in murein biosynthesis through independent pathways [27]. Cell division is coordinated with chromosome separation with the help of the FtsK/SpoIIE family of DNA translocases [28]. The division site selection is greatly influenced by Nucleoid Occlusion (NO) and the Min proteins. The NO protein factors *noc* in *B. subtilis* and *slmA* in *E. coli* prevent the assembly of the divisome near the nucleoid [29,30]. An FtsZ-inhibitor protein, MinC, and its activator MinD are recruited to the mid-cell and also to the cell poles via interaction with MinJ and DivIVA in *B. subtilis* [31]. In contrast, the position of Z-ring assembly in *E. coli* is determined by an ‘oscillatory’ motion of the MinCDE complex [32]. FtsZ then assembles onto the mid-cell site with help of membrane-associated proteins. In *B. subtilis* it is FtsA, while *E. coli* requires dual membrane anchors, ZipA and FtsA [16,33,34]. EzrA and SepF, only identified in gram-positive bacteria, also play a role in the membrane association of FtsZ [35,36]. Throughout the division process, FtsZ interacts with several proteins classified as early and late divisome proteins. Early divisome proteins in *B. subtilis* that interact with FtsZ include FtsA, ZapA, SepF, and EzrA [37]. EzrA, SepF, and ZapA have been recently shown to be essential for the condensation of FtsZ filaments into the Z-ring [38]. Late divisome proteins include GpsB, FtsL, DivIB, FtsW, Pbp2B, and DivIVA [37]. In *E. coli*, FtsA, FtsE, and Zap proteins form the early divisome proteins and during later stages of cell division, FtsZ interacts with FtsK, FtsQ, FtsL, and FtsB, followed by FtsW with FtsI [39–41]. Polymerization of FtsZ and formation of the Z-ring are controlled by the tight regulation and coordination between early and late divisome proteins. The FtsZ polymers undergo treadmilling around the Z-ring to distribute the PG synthases forming the septum [4,5,42]. The rate of FtsZ treadmilling directly influences the septum PG distribution in both *E. coli* and *B. subtilis* and septum synthesis only in *B. subtilis* [4,5]. The difference is attributed to the latter’s requirement for a thicker cell wall [43]. The activation of cell wall synthesis drives septum formation and membrane invagination. It was recently shown that early divisome protein, FtsA interacts with late divisome proteins such as FtsW to activate septal PG synthesis [44]. Final constriction at the septum results in cytokinesis leading to the separation of the daughter cells.

### Cocci have a single coordinator—the divisome complex

A gram-positive coccus, *S. aureus*, is an opportunistic pathogen which is a leading cause of bacteremia and infective endocarditis. The treatment of these infections, along with serious skin and soft tissue infections it causes, is threatened by a rising number of multi-drug resistant strains of the bacterium [45]. Multi-drug resistant *S. aureus* (MRSA) evades antibiotic treatment by synthesizing β-lactamase and PBP2a, which confer the resistance to β-lactam antibiotics [46].

In the cell division of cocci like *S. aureus*, the striking difference with model rods is the absence of an elongation machinery or MreB homologs (Figure 1B). The PG synthesis takes place mostly at the site of the division, i.e., the septum coordinated by FtsZ, leading to orthogonal division over several cell division cycles [47–49]. FtsW-PBP1 and RodA-PBP3 pairs are reported to regulate the septal and lateral PG synthesis, respectively [50]. Another recently discovered protein conserved in the family is SmdA, bearing roles in cell division and cell morphology [51]. In the absence of the Min system, the Noc system plays the role in the division site selection [52]. An early divisome protein GpsB from *S. aureus* was reported to have a direct cell division-modulating activity, unlike the homolog in *B. subtilis* [53]. It was recently shown to also regulate the wall teichoic acid biosynthesis [54]. CozEa and CozEb proteins, which have a homolog in *S. pneumoniae*, are found to interact with EzrA and regulate cell division [55]. Cytokinesis was proposed to be biphasic with a second, faster constriction process at the septum being independent of FtsZ function and likely dependent on PG deposition [56]. The septum formation takes place in a centripetal manner without elongation of the bacteria till the walls are pinched off after constriction. Immediately, the septum is reshaped into a curved hemisphere to give two daughter cocci [57,58]. The switch from PG synthesis at the cell periphery to the septum for a new cycle of cell division is directed by the lipid transporter protein MurJ [56]. This transition would take place after the Z-ring assembly and initiation of cell division, when MurJ is recruited by the late divisome complex [56].

### The in-between bacterial morphology: ovococci

Another opportunistic pathogen *S. pneumoniae*, a gram-positive ovococcoid bacterium, resides in the human respiratory tract. It causes potentially life-threatening infections like pneumonia and meningitis. More than a hundred serotypes of the bacterium are discovered so far, contributing immensely to the growing anti-microbial resistance problem. Like *S. aureus*, horizontal gene transfer in *S. pneumoniae* has allowed the bacteria to develop mutations in PBP’s thereby evading antibiotics [59]. Several recent reports on *S. pneumoniae* cell division have contributed to the understanding of ovococci morphology [60–62].

In *S. pneumoniae*, the cell division time takes longer than the rods, approximately 30 min and the dividing cells mostly exist in pairs (also called diplococci) [63]. Min and Noc systems for division site selection and MreB homolog for directing the elongation are all absent in *S. pneumoniae* [60]. The striking feature of this ovococcoid bacterium is that the cell division cycle begins even before the completion of the previous cell division cycle [62,64]. The nascent equatorial rings are formed at the mid-cell region of the daughter cells even before they are separated from each other, over the undivided nucleoid (Figure 1C). This seemingly overlapping process of cell division cycles may lead to chains of dividing bacteria in a ‘streptococci’.

A mid-cell anchored protein, MapZ is positioned at the mid-cell by the new peptidoglycan synthesis acting as a guide for the movement of FtsZ and its associating proteins, from the septal ring to the equatorial ring to initiate a new cycle of cell division [65–67]. It was recently discovered that CcrZ is responsible for the regulation of DNA replication and the regulation of FtsZ and the Z-ring during cell division initiation [68]. FtsZ associating proteins FtsA and EzrA are essential for *S. pneumoniae*, being part of the initiation complex localizing with FtsZ throughout the cell division process [67,69]. It has been suggested that the role of EzrA in Z-ring assembly and PG synthesis might be central to overall cell division in *S. pneumoniae* [69]. ZapA and its interacting partner ZapJ are also shown to be FtsZ-regulators [69]. FtsZ treadmilling drives septal PG synthesis in concert with the FtsW-PBP2x complex [67]. The rate of FtsZ treadmilling and septal PG synthesis rate are independent, unlike the case with *B. subtilis* [67]. The position of the peripheral PG synthesis in *Streptococci* is different to that of these rods—it is positioned at the septal region and not at the side wall [70]. The two complexes for septal and peripheral PG synthesis assemble at the mid-cell region, and during division form concentric circles, with the septal PG synthesis taking place at the inner closer ring while peripheral PG synthesis takes place on the outer ring [71]. The peripheral elongation of *S. pneumoniae* is carried out by the elongasome complex. This complex, driven by the RodA-PBP2b pair of proteins, consists of CozE, MreC, MreD, and RodZ [60,61,72]. Out of the many models that describe the ovoid pneumococcal division, evidence supports the model where the two complexes for peripheral and septal peptidoglycan synthesis are assembled at the mid-cell forming one large protein complex driving both growth and division of *S. pneumoniae*.

### An atypical form of bacterial cell division: asymmetric cell division

One of the most notorious pathogens is *M. tuberculosis*, an intracellular acid-fast rod. *M. tuberculosis* causes tuberculosis which is the leading cause of infection-related deaths with a death toll of 1.5 million each year [73]. *M. tuberculosis* is also associated with several pulmonary diseases and auto-immune disorders like sarcoidosis and lung cancer [74]. Apart from the absence of the common virulence factors and the latency period inside the host cells, the treatment of this highly adaptable pathogen is challenged by growing antimicrobial resistance. A possible reason for the requirement of longer periods of antibiotic treatment is the asymmetric cell division exhibited by the bacterium making the population heterogeneous [75].

Even though *M. tuberculosis* is a rod-shaped bacterium, it has some distinct features which have resulted in the knowledge gap in understanding its cell division. A key characteristic feature of *Mycobacterial* cells is the cell wall composed of mycolic acid, arabinogalactan, and the PG, which makes it a very thick layer [76]. Typically requiring approximately 18–24 h to divide, *M. tuberculosis* requires a special cohort of proteins that enable its division which is very different than typical rods *B. subtilis* and *E. coli* [77,78]. The geometry of cell division is asymmetrical (Figure 1D) exhibited by a polar growth, i.e., it takes place mostly at the poles of the bacterium rather than the side walls [75–78]. Unlike in *B. subtilis* where DivIVA regulates septum formation, its homolog in *M. tuberculosis*, Wag31 directs and regulates the polar growth [79]. Recent discovery revealed that a protein LamA maintains the asymmetry in mycobacteria and interacts with Wag31 [80]. In the absence of the MreB homolog, several pieces of evidence suggest that the timing of elongation and division machinery may be independent of each other [77,81]. The division site for Z-ring assembly is not regulated by the Noc or the Min system, as they are both absent in *Mycobacteria*. The division site is suggested to be present as troughs within the cell surface that have been inherited from one or two cell division cycles prior [82]. SepF is the only early divisome protein known to interact with FtsZ in *M. tuberculosis*, even though several late divisome proteins such as FtsW, showing a significant role in mycobacterial cell division, are present similar to *B. subtilis* [77,83,84]. FtsA and ZipA are both absent in *M. tuberculosis*, with studies proposing that FtsW might substitute their roles within this bacterium [84,85]. Recent studies have shown that a transmembrane protein unique to the family, CrgA, interacts with FtsZ and plays an important role in several steps of the cell division process [86]. CrgA also shows interactions with CwsA, a protein specific to mycobacteria that regulates Wag31 functioning [87]. WhmD and Rv0954 are other proteins that interact with the Z-ring and/or regulate the cell cycle in the mycobacterium species [88–90]. Cell wall deposition by the PBPs and their partners happens at a faster rate at the one pole, indicated as the old pole, inherited from the mother during the previous cell cycle [76]. Finally ‘V-snapping’ of the septum divides the cell into two, with the daughter cells of uneven sizes-the old-poled daughter bigger than the new-poled daughter cell [76–78]. The new cell cycle begins with the newly separated cell wall becoming the new poles of the daughter cells. Asymmetrical cell division gives the bacteria a survival advantage due to heterogeneity in the population, in the absence of which the bacteria is more susceptible to antibiotics [80].

Asymmetrical cell division is observed in other bacteria as well. It was recently reported that in gram-negative bacteria that exhibit asymmetrical division, short filaments of FtsZ were enough to begin the constriction process rather than the assembly of the entire Z-ring, unlike observed in other asymmetrically dividing bacteria [91].

## Twist in the tail: dissimilar c-terminal tails of FtsZ from different bacteria

### Structure of FtsZ

**Figure 2:**
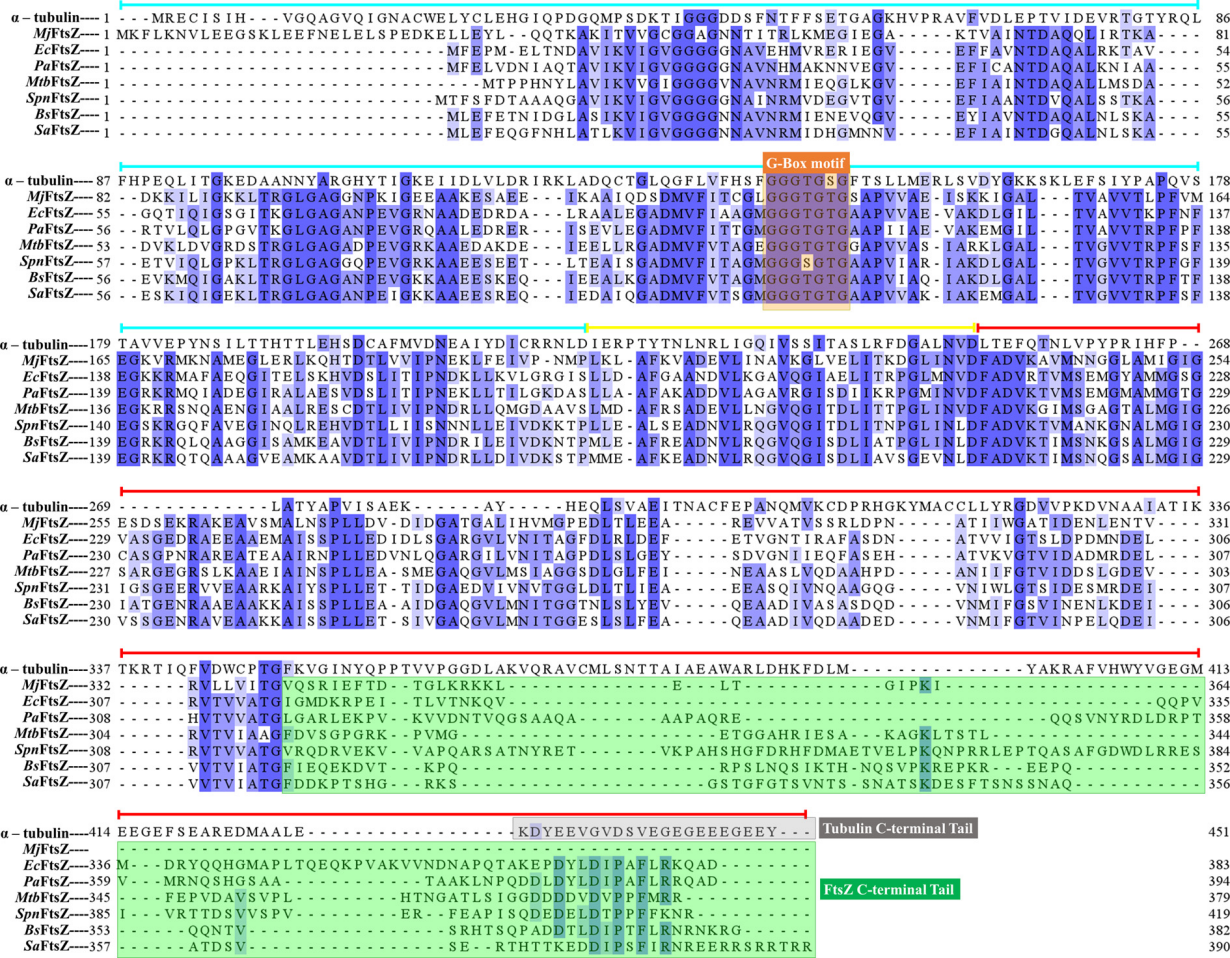
Sequence alignment of FtsZ and eukaryotic tubulin Clustal Omega tool was used to align the amino acid sequences of human α-tubulin isoform 1A (α-tubulin, 451 residues), *M. jannaschii* FtsZ (*Mj*FtsZ, 364 residues), *E. coli* FtsZ (*Ec*FtsZ, 383 residues), *P. aeruginosa* FtsZ (*Pa*FtsZ, 394 residues), *M. tuberculosis* FtsZ (*Mtb*FtsZ, 379 residues), *S. pneumoniae* FtsZ (*Spn*FtsZ, 419 residues), *B. subtilis* FtsZ (*Bs*FtsZ, 382 residues), and *S. aureus* FtsZ (*Sa*FtsZ, 390 residues). The residues that show identity are marked in blue using Jalview. N-terminal and C-terminal domains separated by the T7 loop region is indicated in blue, red, and yellow lines, respectively, above the sequence alignment. The G-box GTP binding motif and the C-terminal tails are highlighted.

**Figure 3:**
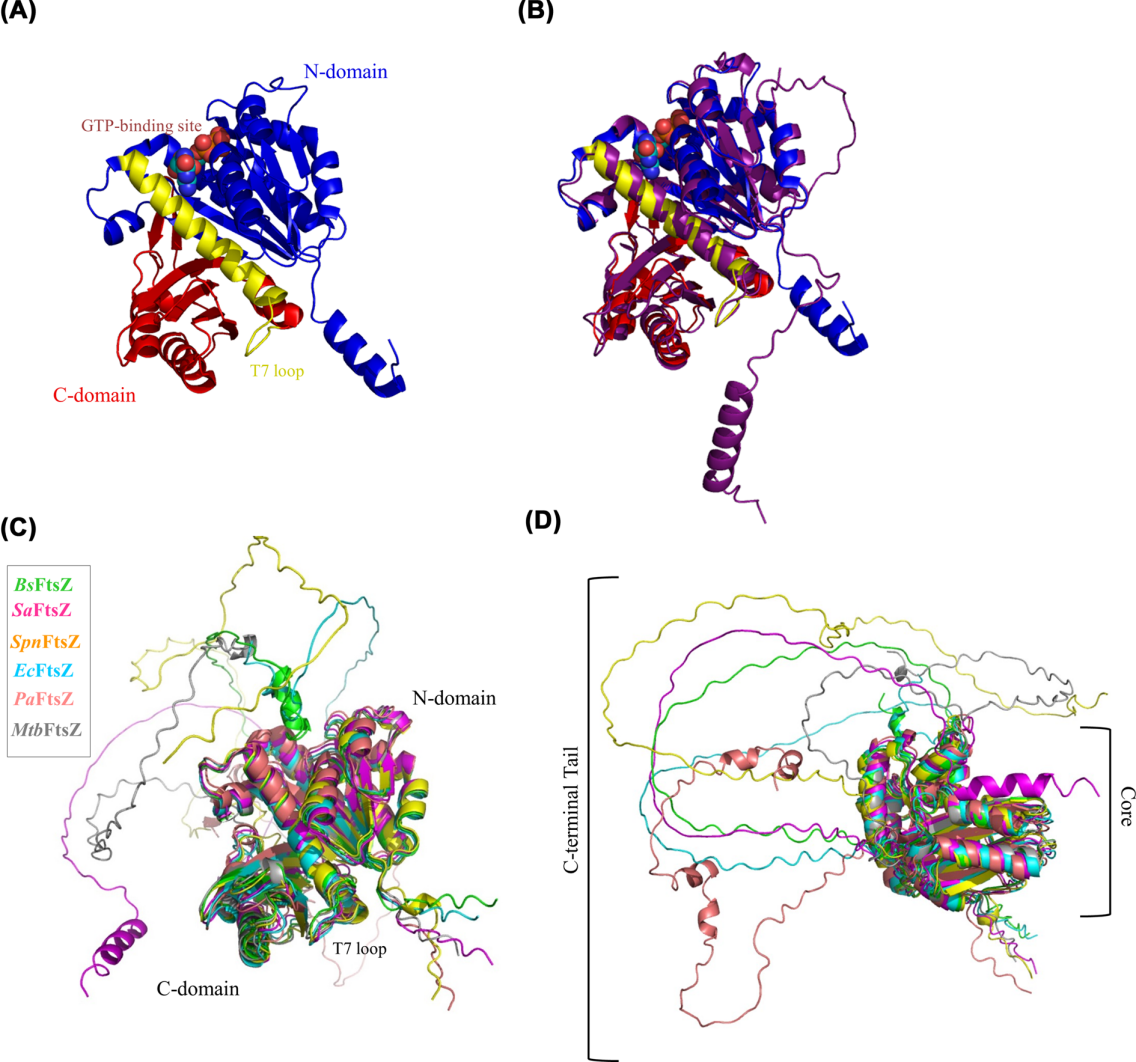
Structure of FtsZ protein (**A**) The crystal structure of *M. jannaschii* FtsZ showing the N-terminal domain in blue and the C-terminal domain in red separated by the H7 helix and T7 loop (yellow). N-terminal domain shows GTP bound to the structure. PDB: 2VAP (**B**) Superimposition of the X-ray crystal structure of *M. jannaschii* 2VAP with AlphaFold prediction for the same shown in purple. (**C**) Superimposition of AlphaFold predictions of *B. subtilis* FtsZ (*Bs*FtsZ) in green, *E. coli* FtsZ (*Ec*FtsZ) in blue, *S. aureus* FtsZ (*Sa*FtsZ) in magenta, *S. pneumoniae* FtsZ (*Spn*FtsZ) in orange, *M. tuberculosis* FtsZ (*Mtb*FtsZ) in grey and *P. aeruginosa* FtsZ (*Pa*FtsZ) in pink. (**D**) Intrinsically disordered C-terminal shown to be highly flexible compared to the compact, highly conserved core region.

As seen in the previous section, FtsZ is the central player in cell division that warrants deeper investigation at the protein level. Amino acid sequence alignment of all the different FtsZs in this review as well as the eukaryotic homolog of FtsZ, tubulin, is shown in Figure 2 [92–94]. FtsZ bears little sequence similarity with tubulin, barring the GTP binding region called the G-box motif [1]. However, the homology between FtsZ and tubulin is established because of the highly similar secondary structures of the two proteins [1]. FtsZ from *Methanocaldococcus jannaschii* was the first FtsZ to be crystallized in 1998 [95]. Very similar to tubulin, the crystal structure of FtsZ shows an N-terminal core domain bearing the GTP-binding site and a C-terminal core domain bearing the T7 loop (Figure 3). Rossmann folding establishes an arrangement of a core of β-sheets alternating with α-helices surrounding it as seen in Figure 3A [96,97]. The H7 helix separates the N- and C-termini cores. The active site is formed when the T7 loop of one FtsZ monomer interacts with the GTP binding domain of the adjacent monomer, forming an end-to-end connection. Single-stranded filament assembly is promoted by the conformational switch from low affinity to higher affinity for polymer formation, which allows the establishment of cooperativity [98–102]. The conformational switch is characterized by the relative rotation of the C-terminal T7 loop by ∼27^o^ with respect to the N-terminal domain, pulling the H7 helix downwards [98,101]. By using tryptophan fluorescence as a detector of the change in the environment of T7 loop region, this conformational switch was recently observed in FtsZ from *S. pneumoniae* [102].

As important domains forming the active site, the N-terminal regions and the C-terminal core domains are highly conserved in FtsZ (Figure 2). This indicates that the FtsZ-FtsZ interaction to form a polymer is conserved for all FtsZ. This makes up for approximately 80% of the residues in the amino acid sequence of FtsZ. However, several assembly-related characteristics arise for FtsZ from different bacteria. These are usually observed in spectroscopy and electron microscopy studies of protofilaments formed *in vitro* [103–106]. Further, functional differences like treadmilling properties and interactions with associated proteins arise, as seen in the previous section. Apart from the cellular factors contributing to these differences, the differences in the FtsZ behavior at the structural level arise from the C-terminal end domain, specifically the much less conserved C-terminal tail region.

### C-terminal tail of FtsZ

With the recent advent of the AI-predicted modeling of protein structures through AlphaFold, it has become easier to visualize these disordered C-terminal tails relative to the defined structure of the core domains [107,108]. To demonstrate the accuracy of the AlphaFold prediction, the crystal structure of FtsZ in Figure 3A is superimposed with the AlphaFold prediction (Figure 3B). For *B. subtilis* FtsZ (*Bs*FtsZ), *E. coli* FtsZ (*Ec*FtsZ), *S. aureus* FtsZ (*Sa*FtsZ), *S. pneumoniae* FtsZ (*Spn*FtsZ), *M. tuberculosis* FtsZ (*Mtb*FtsZ), and *P. aeruginosa* FtsZ (*Pa*FtsZ), AlphaFold predictions are used (Figure 3C). Superimposition of the structures shows that the core domains of N- and C-termini show highly conserved structural similarity. The differences arise in the disordered C-terminal tail (CTT) region (Figure 3D).

**Figure 4:**
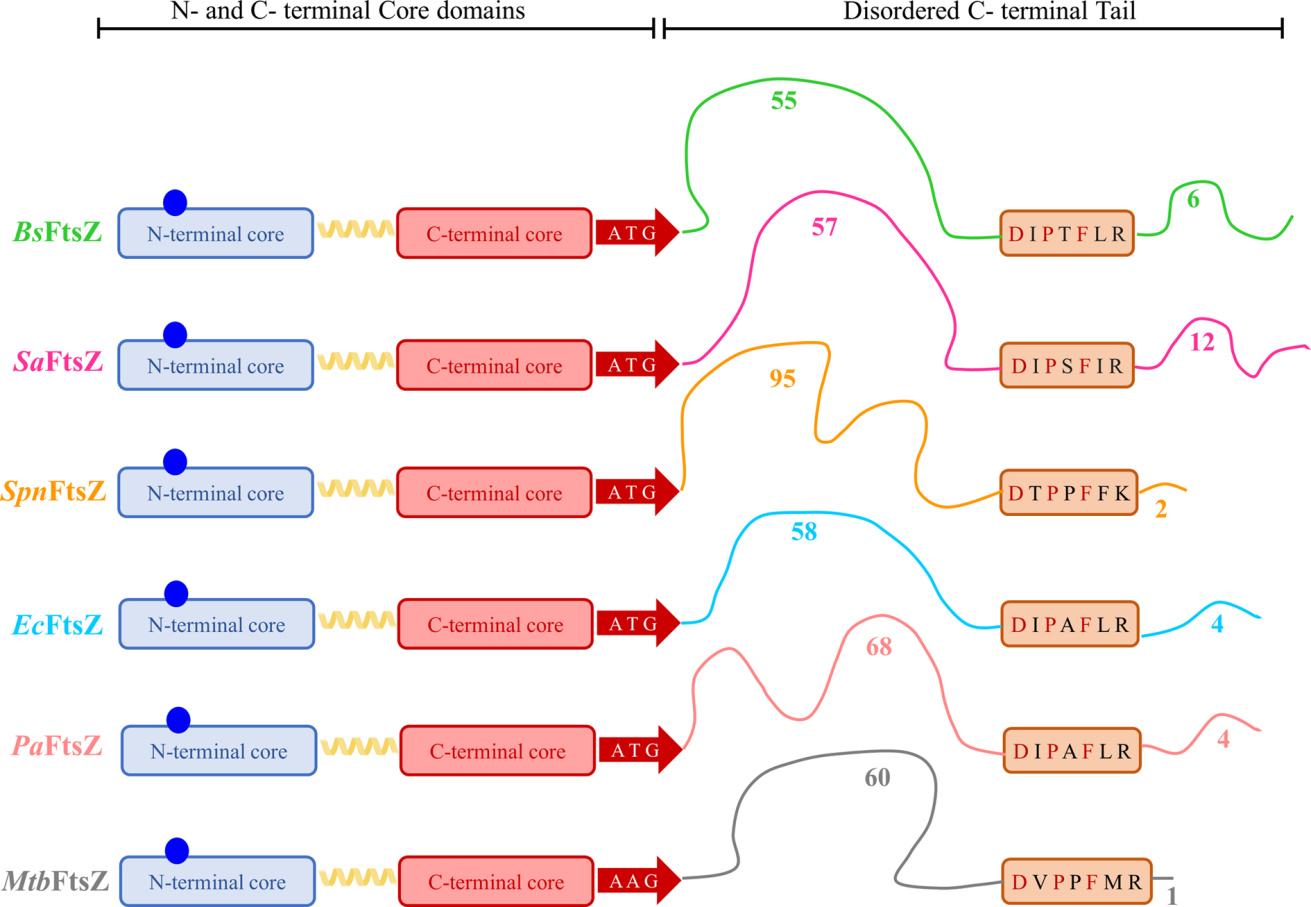
Schematic of conservation of FtsZ sequence and structure in model organisms and pathogens The sequence and structural similarity between *B. subtilis* FtsZ (*Bs*FtsZ) in green, *E. coli* FtsZ (*Ec*FtsZ), *S. aureus* FtsZ (*Sa*FtsZ), *S. pneumoniae* FtsZ (*Spn*FtsZ), *M. tuberculosis* FtsZ (*Mtb*FtsZ), and *P. aeruginosa* FtsZ (*Pa*FtsZ) are compared. The core region of FtsZ is composed of a conserved N-terminal domain with a GTP (Shown as a blue circle) binding region and the C-terminal core region separated by the H7 helix. The intrinsically disordered tail begins after ‘ATG’ stretch of amino acids around 308-312 in the sequence. This intrinsically disordered region is composed of the C-terminal linker (CTL) followed by the C-terminal conserved (CTC) and C-terminal variable (CTV) regions. The CTL and CTV of different FtsZ are of variable length and show very less sequence similarity with each other.

A comparison of the FtsZ sequence and structure between the model rods and the four pathogenic organisms is shown in Figure 4. The disordered highly variable region starts after the ‘ATG’ peptide which occurs at the 308-312 residues number in the amino acid sequence for the FtsZs considered. Sequence alignment shows that the sequences up to ‘ATG’ show more than 60% similarity. The C-terminal tail region is variable in length ranging from 68 to 104 residues (Figure 4). This CTT region has less than 20% similarity between the sequences. The CTT region is made up of a long C-terminal linker (CTL) region, a conserved C-terminal conserved peptide (CTC) and a C-terminal variable (CTV) region. The CTC region is an 8-residue stretch with fully conserved aspartic acid, proline, and phenylalanine residues. The rest of the 5 residues show more than 50% similarity between the organisms. The CTV region has 1-10 residues, with a net positive charge in gram-positive organisms and a net neutral charge in gram-negative organisms.

### Structure of the C-terminal tail of FtsZ

Even though several crystal structures of FtsZ have been so far elucidated, the C-terminal end region has not been modeled through crystallography, indicating its disordered nature. However, several groups have shown that binding of the CTC with several FtsZ-associated proteins stabilizes the region to different conformations. From adopting a β-strand and helical conformation upon binding to ZipA [109], an extended conformation when bound to SlmA [100] and SepF [110], to interacting with FtsA [111] and ZapD [112] in a helical conformation, X-ray crystal structures of FtsZ bound to several accessory proteins shows different conformations of the CTC region. However, without any protein binding to this linker region, CTT remains an intrinsically disordered region, as recently shown by NMR spectroscopy of *E. coli* FtsZ [113]. Replacing the disordered region with rigid conformations results in slower assembly of FtsZ and aberrant GTPase activity emphasizing that the unstructured nature of the CTT is critical for its function [114].

### The function of the C-terminal tail of FtsZ

The flexible CTL is shown to be important for the protofilament formation and lateral interactions of FtsZ *in vitro*, and for FtsZ’s attachment to the membrane and proper cell division *in vivo* [114–116]. While the sequence of this disordered region did not affect the functioning of FtsZ, the flexibility, net charge, and length were important factors for CTL function [114]. As seen for *Ec*FtsZ, any disordered peptide of the length between 43 and 95 amino acids forms a viable linker, while longer or shorter peptides do not allow cell division to occur [115]. Increasing the linker peptide length to more than 100 residues in *Bs*FtsZ also leads to perturbation of position of Z-ring and frequency of cell division [114]. Studies indicate that CTL might be linked to the generation of force during constriction and membrane invagination [116]. However, the exact role of the CTL in FtsZ function remains elusive.

While the CTL acts as a ‘spacer’ region for higher-order assemblies of FtsZ, the CTC and CTV stretch acts as a ‘sticker’ for interactions of the C-terminal tail with the core [117,118]. CTL shows auto-inhibitory functions preventing higher-order assemblies of FtsZ and the CTC and CTV stretch of *Bs*FtsZ were shown to be important for the autoregulation of GTP hydrolysis and polymerization [117]. Specifically, the CTV was indicated for an important role in lateral interactions and bundling of *B. subtilis* FtsZ protofilaments *in vitro*, the deletion of which results in disrupted cell division [119]. The net charge of CTV also seemed to affect the bundling propensity of the protein [119]. However, the same effect was not observed for *E. coli* FtsZ, which bears a neutrally charged CTV [119]. The significance of this difference in the net charge of CTV of FtsZ in gram-positive and gram-negative organisms remains to be explored.

The CTC and CTV regions are together called the ‘grappling hook peptide’ due to their association with other proteins of the divisome in the cytoplasm and the plasma membrane [18,114]. Proteins that are associated with the positioning of the Z-ring, such as MinC [120–122], SlmA [29,123,124], and MapZ [125] are shown to bind to this extreme C-terminal region of FtsZ. This region also enables the binding of early divisome proteins, FtsA [126–128], EzrA [129,130], SepF [131], ZipA [109,132–134], and the Zap proteins [135,136] to FtsZ.

*Spn*FtsZ has an unusually long C-terminal tail with 104 residues (Figure 4). Considering that FtsZ binds to EzrA and FtsA throughout the cell cycle [69] and also binds to MapZ during the initiation of cell division [65], one can speculate that the longer length may be required to accommodate all these proteins bound to FtsZ C-terminal peptide at the same time. As shown in a visual review of *S. pneumoniae* [61], visualization of the arrangement of the proteins within the Z-ring and their interactions may help to understand the significance of the C-terminal linker lengths and flexibility. It would be interesting to determine whether this C-terminal region and its binding to FtsZ-interacting partners have any influence on the timing of cell division and establishment of pathogenicity/virulence of the bacteria.

### Insights from C-terminal tails of eukaryotic tubulin

Little is known about the C-terminal tail of FtsZ, compared with the C-terminal tail of tubulin, a region that is also disordered and highly variable within various tubulin isoforms. Unlike FtsZ, CTT of tubulin is usually shorter in length with 10–20 residues across isoforms and is highly negatively charged (Figure 2) [137]. The variations within the CTT of tubulin are established for fine-tuning of different biochemical functions of the protein without disturbing the core that is essential for polymerization [137]. CTT is a region important for post-translational modifications of the protein that establishes the regulatory mechanism for microtubule binding motors [137–139]. Whether FtsZ’s C-terminal tail plays any such role in the regulation of the bacterial cell cycle remains to be explored. However, like the CTT of FtsZ that binds to FtsZ-associated proteins, the C-terminal tails of tubulin are also important for the binding of several microtubule-associated proteins. In the case of tubulin, the conformations of the CTT region bound to different proteins remain largely unexplored [140]. Cues from the behavior of CTT from tubulin and FtsZ would aid in understanding the biological functions of this versatile and significant region of polymer proteins.

## Inhibiting ‘divide and rule’: anti-FtsZ agents for anti-bacterial therapy

### Inhibition of cell division and FtsZ as an alternative strategy for anti-bacterial therapy

**Figure 5:**
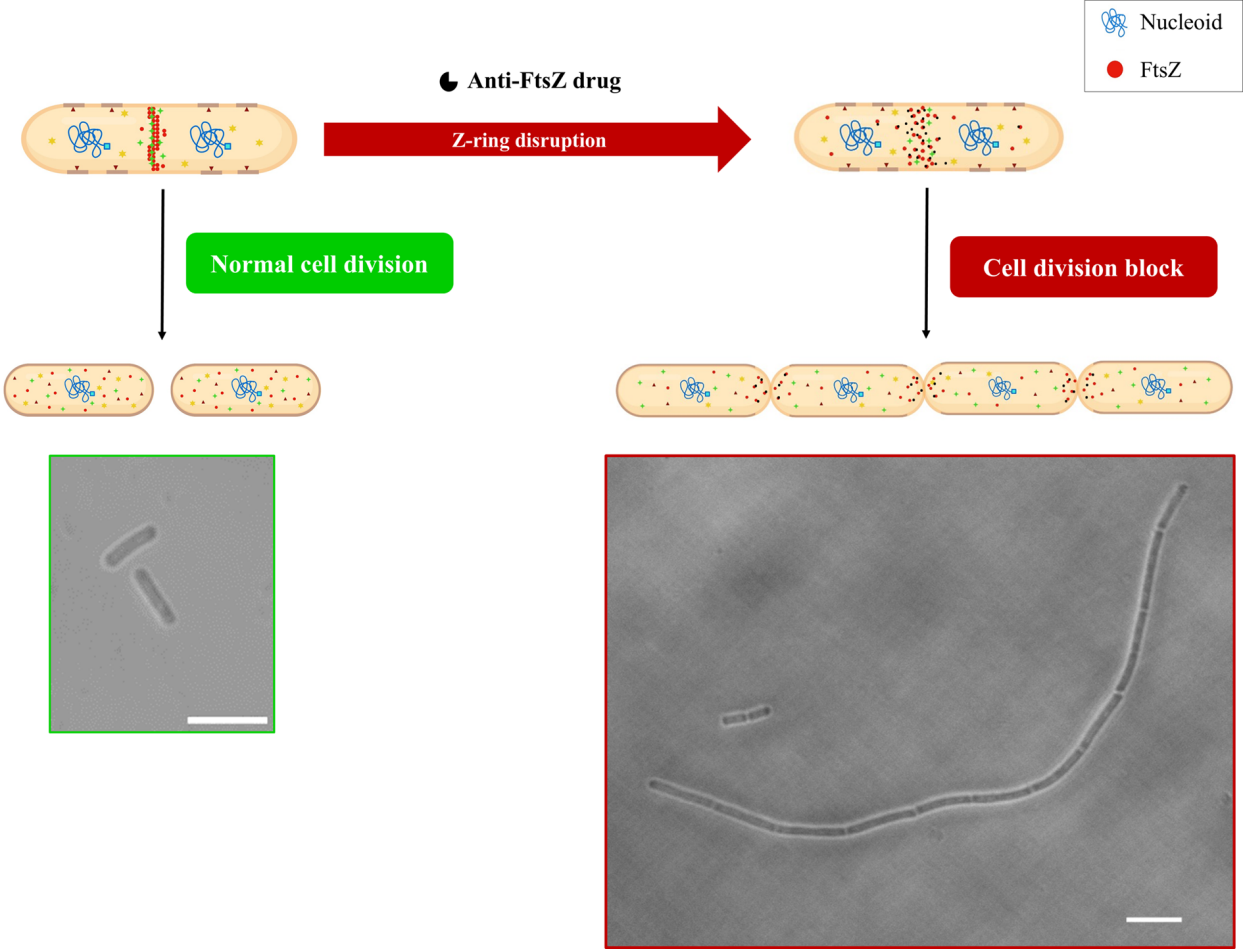
Targeting cell division and FtsZ for anti-bacterial therapy Z-ring dynamics during normal cell division drive the successful separation of daughter cells after cytokinesis. The inhibition of FtsZ by anti-bacterial agents leads to the disruption of the Z-ring dynamics, finally leading to cell division block usually visualized as a filamentous phenotype of the elongated but undivided cells. DIC images of *Bacillus subtilis* 168 cells bear scale bar of 5 μm (unpublished data) (Cartoon images were created using BioRender.com)

The disruption of the tight regulation of cell division in bacteria has been identified as an attractive strategy in the discovery of novel antibiotics. One of the highly sought-after targets is FtsZ, the inhibition of which leads to the cell division block. As described above, FtsZ is at the forefront of a plethora of interactions throughout the cell division process. Anti-FtsZ agents would disrupt FtsZ assembly leading to the collapse of the Z-ring, associated protein interactions, and peptidoglycan assembly. In the absence of a proper cell division machinery, the cells become defective after several rounds of elongation without division, a phenotype known as filamentous cells (Figure 5), and finally lyse.

**Figure 6:**
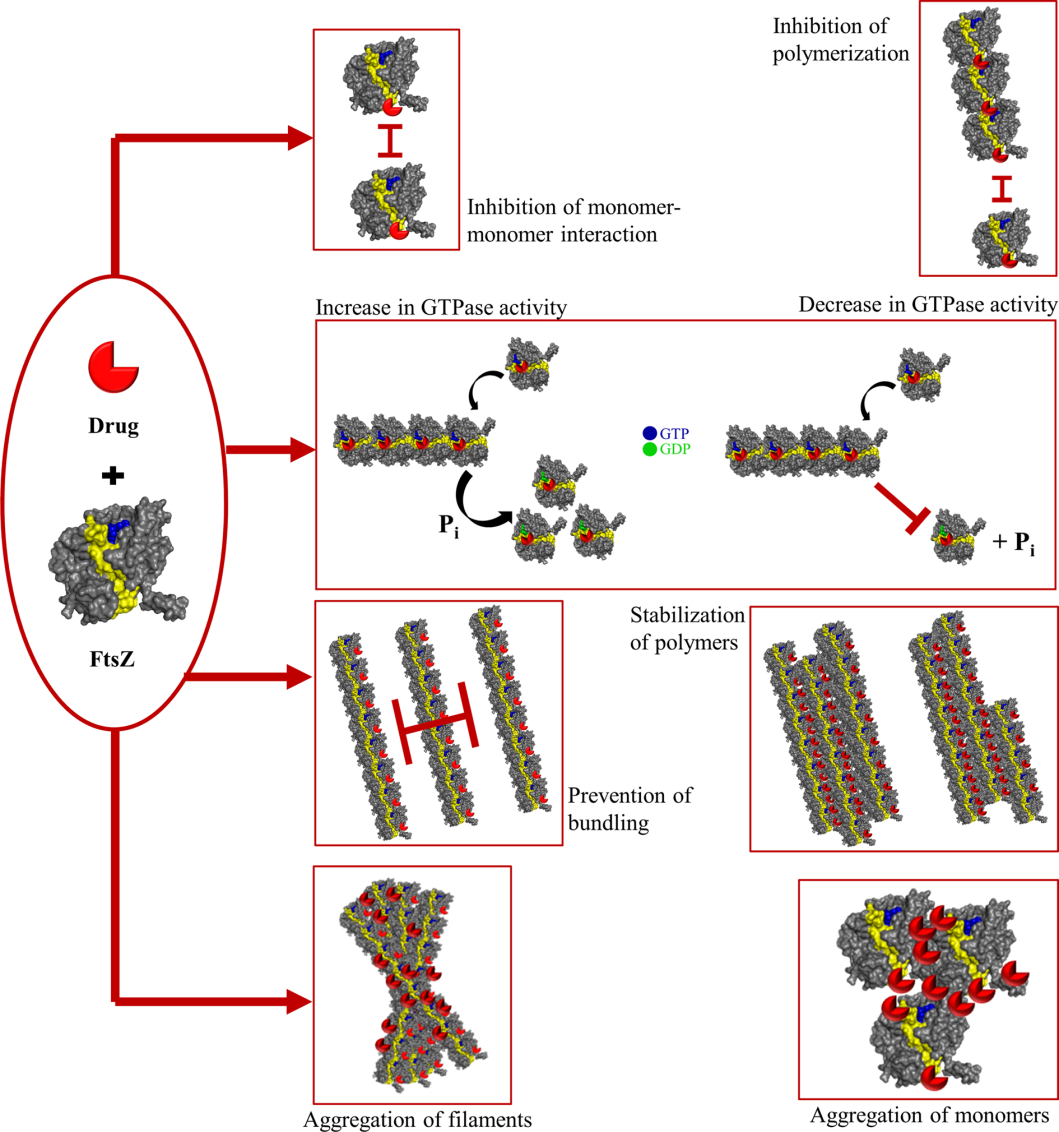
Mechanism of action of anti-FtsZ drugs Anti-FtsZ agents can affect FtsZ in one or more of the following ways: (i) disrupting the monomer-monomer interaction at the active site and inhibiting polymerization, (ii) increasing or decreasing GTPase activity leading to a reduced net addition of monomers on to the polymer, (iii) inhibiting lateral interactions preventing bundling of protofilaments or promoting the lateral interactions and stabilize the polymers, and (iv) inducing aggregation of filaments or of monomers.

Recent research on the small molecules that bind to FtsZ and inhibit its functioning through various mechanisms, has shown that targeting FtsZ is a highly useful strategy for anti-bacterial therapy. Broad-spectrum antibiotics can be developed against FtsZ, one of the most conserved proteins in bacteria. Several excellent reviews have enumerated the vast number of anti-FtsZ agents discovered and their mechanism of action [6,7,11,15,18,141–145]. A schematic of the different effects of anti-FtsZ agents on FtsZ assembly is depicted in Figure 6.

### Binding pockets of FtsZ

**Figure 7:**
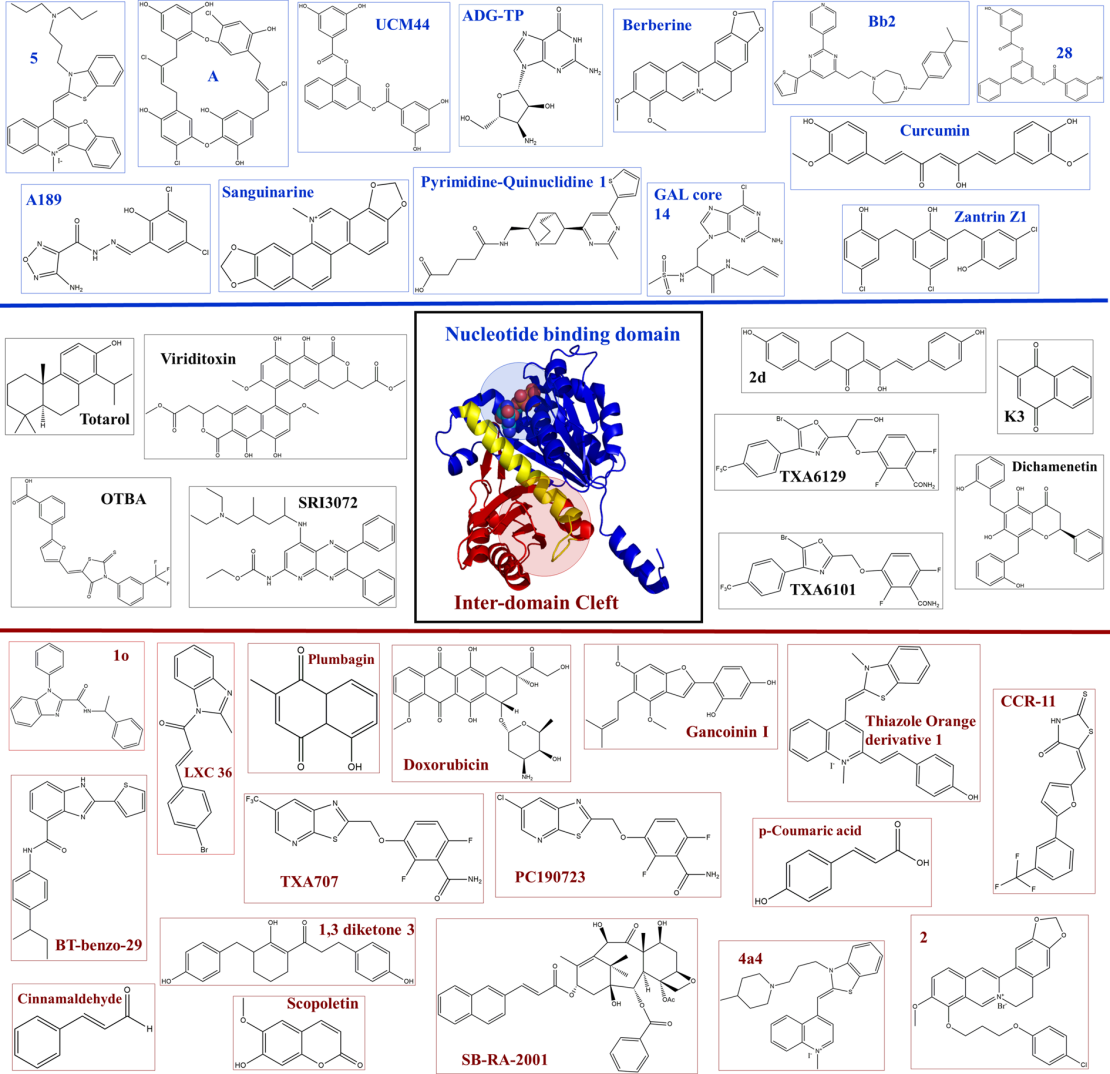
Chemical structures of anti-FtsZ agents Chemical structures of natural and synthetic anti-bacterial agents that target FtsZ have been labeled in blue for those that bind near the nucleotide-binding pocket and red for those that bind near the inter-domain cleft and the T7 loop. The structures labeled in black are given for those compounds whose binding site on FtsZ is not determined yet. Some labels are shortened denotations from Table 1. For further descriptions of the compounds please see Table 1.

FtsZ has two important binding pockets that have been identified [1,21]. The nucleotide-binding pocket and the inter-domain cleft are shown in Figure 7. Due to the similarity of the nucleotide-binding pocket of FtsZ to that of the eukaryotic tubulin, it is more advantageous to design inhibitors to FtsZ that bind to the inter-domain cleft [1,21]. It is to be noted that during polymerization these two regions come in close contact with each other forming the active site. This indicates that the surrounding areas of the protein are yet to be explored and targeted by small molecules.

Along with their structures provided in Figure 7, Table 1 provides a list of anti-FtsZ agents discovered, particularly with activity against pathogens *S. aureus, S. pneumoniae, M. tuberculosis, P. aeruginosa*, and gram-negative bacteria. The anti-FtsZ agents have been divided according to their binding sites as nucleotide-binding domain binders, inter-domain cleft binders, and unknown (for those drugs that have no information regarding their binding site available).

**Table 1:** List of anti-FtsZ agents with their source, binding region, mode of action, and activity on bacteria

Source	Anti-FtsZ agent	Mode of action on FtsZ	Anti-bacterial activity	References
**Nucleotide-binding domain binders**				
Plant-derived	Sanguinarine	Inhibits bundling and assembly of *Ec*FtsZ protofilament	***B. subtilis*** IC_50_: 3 ± 1 µM ***E. coli*** IC_50_: 14 ± 2.3 µM	[175]
	Berberine	Inhibits assembly and GTPase activity of *Ec*FtsZ and *Sa*FtsZ	***S. aureus*** MIC: 128 µg/ml ***B. subtilis*** MIC: 128 µg/ml ***E. coli*** MIC: N/A	[176,177]
	Curcumin	Increases GTPase activity of *Ec*FtsZ	***E. coli*** MIC_80_: 100 µM ***B. subtilis*** MIC:100 µM	[178]
Algae-derived	Chrysophaentin **A**	Inhibits polymerization and GTPase activity of *Ec*FtsZ, *Bs*FtsZ, and *Sa*FtsZ	***S. aureus*** MIC: 2 µg/ml ***B. subtilis***, ***E. coli*** MIC: N/A	[179–181]
Gammaproteo-bacteria	Aminodeoxyguanosine triphosphate **(ADG-TP)**	Inhibits *Ec*FtsZ polymerization and its localization during cell division	***E. coli*** MIC: 4–16 µg/ml ***Klebsiella pneumonia*** MIC: 16 µg/ml	[182]
Synthetic compounds	GAL core compound 14	Inhibits GTPase activity of *Pa*FtsZ	***P. aeruginosa*** IC_50_: 0.6 mM ***S. aureus*** IC_50_: 0.6 mM	[183]
	4-Aminofurazan derivative **A189**	Inhibits assembly of *Ec*FtsZ and cell division	***E. coli*** MIC: 64 µg/ml ***S. aureus*** MIC: 16 µg/ml	[184]
	Zantrin (Z1)	Perturbs *Ec*FtsZ assembly	***E. coli*** MIC: 20 µM ***B. subtilis*** MIC: 1.25 µM ***P. aeruginosa*** MIC; 40 µM ***S. pneumoniae*** MIC: 0.312 µM ***S. aureus*** MIC: 2.5 µM	[185]
	UCM44	Enhances *Bs*FtsZ assembly	***B. subtilis*** MIC: 16 mg/L ***S. aureus*** MIC: 16 mg/L ***S. pneumoniae*** MIC: 32 mg/L ***P. aeruginosa*** MIC: 64 mg/L ***E. coli*** MIC: 128 mg/L	[153]
	Benzofuroquinolinium derivative **5**	Inhibition of GTPase activity and polymerization of *Ec*FtsZ	***B. subtilis*** MIC: 0.25 µg/ml S. aureus^*^ MIC: 0.25–1 µg/ml ***E. coli*** MIC: 1 µg/ml ***P. aeruginosa*** MIC: 4 µg/ml	[186]
	Pyrimidine-quinuclidine derivative **1**	Inhibits GTPase activity of *Sa*FtsZ	***S. aureus*** MIC: 897.1 µM ***E. coli*** MIC: 449 µM	[187]
	2,4-Disubstituted-6-thiophenyl- pyrimidine **Bb2**	Inhibits polymerization and GTPase activity of *Sa*FtsZ	***B. subtilis*** MIC: 2 µg/ml ***S. aureus*** MIC: 2 µg/ml ***E. coli*** MIC: 128 µg/ml	[188]
	Biphenyl derivative **28**	Inhibits filament dynamics of *Bs*FtsZ	***B. subtilis*** MIC: 5 µM ***S. aureus*** MIC: 7 µM	[189]
**Inter-domain cleft binders**				
Plant-derived	Cinnamaldehyde	Inhibits polymerization and GTPase activity of *Ec*FtsZ	***E. coli*** MIC: 1000 mg/L ***B. subtilis*** MIC: 500 mg/L ***S. aureus*** MIC: 250 mg/L	[190]
	Plumbagin	Inhibits assembly and GTPase activity of *Bs*FtsZ	***B. subtilis*** MIC: 29 µM ***M. smegmatis*** MIC: 31 µM	[147]
	Scopoletin (coumarin)	Inhibits polymerization and GTPase activity of *Ec*FtsZ	***M. tuberculosis*** MIC: 42 µg/ml ***E. coli*** MIC: N/A	[191,192]
	Gancaonin I (polyketide)	Inhibits GTPase activity of *Bs*FtsZ	***B. subtilis*** MIC: 5 µM	[193]
	p-Coumaric acid	Inhibits GTPase activity of *Ec*FtsZ	***S. pneumoniae*** MIC: 20 µg/ml ***S. aureus*** MIC: 20 µg/ml ***B. subtilis*** MIC: 20 µg/ml ***E. coli*** MIC: 80 µg/ml	[194,195]
Actinobacterium	Doxorubicin	Inhibits assembly and GTPase activity of *Ec*FtsZ	***E. coli*** MIC: 20 µM ***B. subtilis*** MIC: 10 µM ***S. aureus*** MIC: 5 µM	[196]
Synthetic compounds	BT-benzo-29	Inhibits GTPase activity and assembly of *Bs*FtsZ	***B. subtilis*** IC_50:_ 1 ± 0.2 μM ***M. smegmatis*** IC_50_: 1.6 ± 0.4 μM	[197]
	PC190723	Induces bundling of *Bs*FtsZ and *Sa*FtsZ and reduces GTPase activity.	S. aureus^*^ MIC: 0.5–1 µg/ml ***B. subtilis*** MIC: 1 µg/ml	[148,149,156]
	TXA707	Enhances *Sa*FtsZ assembly	S. aureus^*^ MIC: 2–4 µg/ml	[158]
	CCR-11	Inhibits assembly and GTPase activity of *Bs*FtsZ	***B. subtilis*** MIC: 3 µM	[143]
	SB-RA-2001	Promote assembly and bundling of *Bs*FtsZ and inhibits its GTPase activity	***M. tuberculosis*** MIC: 60 μM ***B. subtilis*** MIC: 38 µM	[198]
	9-Phenoxy Berberine derivative **2**	Inhibits GTPase activity and polymerization of *Sa*FtsZ	***B. subtilis*** MIC: 4 µg/ml ***S. aureus*** MIC: 2 µg/ml ***E. coli*** MIC: 32 µg/ml	[177]
	Thiazole Orange Derivative **1**	Inhibits GTPase activity and increases the bundling of *Sa*FtsZ and *Ec*FtsZ protofilament	***B. subtilis*** MIC: 1.5 µg/ml S. aureus^*^ MIC: 1.5–3 µg/ml ***E. coli*** MIC: 3 µg/ml ***P. aeruginosa*** MIC: 6 µg/ml	[199]
	1,3 diketone 3	Inhibits *Spn*FtsZ polymerization and induces its aggregation	***B. subtilis*** MIC: 11 µM ***S. pneumoniae*** MIC: 2 µM	[200]
	Thiazole-quinolinium derivative **4a4**	Enhances *Sa*FtsZ assembly	S. aureus^*^ MIC: 1–2 µg/ml ***B. subtilis*** MIC: 1 µg/ml E. coli^*^ MIC: 0.5–1 µg/ml ***P. aeruginosa*** MIC: 64 µg/ml	[201]
	Benzo[d]imidazole-2- carboxamide derivative **1o**	Inhibits the polymerization of *Bs*FtsZ	***B. subtilis*** MIC: 1.56 µg/ml ***M. tuberculosis*** H37 MIC: 0.78 µg/ml	[202]
	Cinnamaldehyde derivative LXC 36	Inhibits GTPase activity and polymerization of *Ab*FtsZ	***A. baumannii*** MIC: 32 µg/ml	[203]
**Unknown binding sites**				
Plant	Totarol	Inhibits assembly and GTPase activity of *Mtb*FtsZ	***B. subtilis*** MIC: 2 µM ***M. tuberculosis*** MIC: 16 µg/ml	[204,205]
Fungal	Viriditoxin	Inhibits polymerization and GTPase activity of *Ec*FtsZ	S. aureus^*^ MIC: 4–8 µg/ml S. pneumoniae^*^ MIC: 2–32 µg/ml ***E. coli*** MIC: >64 µg/ml	[206]
Synthesized (but naturally occurring)	Vitamin **K3**	Increases GTPase activity and inhibits the polymerization of *Spn*FtsZ	***B. subtilis*** MIC: 35 µM ***S. pneumoniae*** MIC: 35 µM	[146]
	Dichamenetin	Inhibits GTPase activity of *Ec*FtsZ	***S. aureus*** MIC: 1.7 µM ***B. subtilis*** MIC: 1.7 µM ***M. smegmatis*** MIC: 3.4 µM ***E. coli*** MIC: N/A	[207]
Synthetic compounds	OTBA	Promotes assembly and bundling of *Ec*FtsZ and *Bs*FtsZ, decreases the GTPase activity	***B. subtilis*** MIC: 2 µM ***E. coli*** MIC: N/A	[208]
	**2d** carbocyclic curcumin	Enhances GTPase activity of *Bs*FtsZ and inhibits the assembly	***B. subtilis*** MIC: 0.7 mg/L ***S. aureus*** MIC: 2 mg/L ***Streptococcus pyogenes*** MIC: 2 mg/L	[209]
	**SRI-3072** (2-alkoxycarbonylamin-o- pyridine)	Inhibits polymerization and GTPase activity of *Mtb*FtsZ	***M. tuberculosis*** MIC: 0.25 mg/L	[210]
	TXA6101	Inhibits *Ec*FtsZ polymerization	***S. aureus*** (MRSA Clinical Isolate MPW020) MIC: 0.125 µg/ml ***E. coli***^†^ MIC: 0.25 µg/mL ***K. pneumoniae***^†^ MIC: 4 µg/ml ***S. sonnei***^†^ MIC: 0.25 µg/ml	[162,163]
	TXA6129	Inhibits *Ec*FtsZ polymerization	***E. coli***^†^ MIC - 0.5 µg/mL ***K. pneumoniae***^†^ MIC: 8 µg/ml ***Shigella sonnei***^†^ MIC: 0.25 µg/ml	[163]

N/A, Not Available.

*Ab* FtsZ, *Acinetobacter baumannii* FtsZ; *Bs*FtsZ, *B. subtilis* FtsZ; *Ec*FtsZ, *E. coli* FtsZ; *Mtb*FtsZ*, M. tuberculosis* FtsZ; *Pa*FtsZ, *P. aeruginosa* FtsZ; *Sa*FtsZ, *S. aureus* FtsZ; *Spn*FtsZ, *S. pneumoniae* FtsZ.

* MIC ranges are mentioned where MIC values for more than one strain of the organism is determined.

† MIC of the compound was determined when used in combination with efflux pump inhibitor PAβN (100 μg mL^−1^).

### Differential effects of anti-FtsZ agents on FtsZ from different bacteria

As seen from Table 1, most of the studies on anti-FtsZ compounds show the mechanism of action demonstrated in the model rods—*B. subtilis* and *E. coli* and their FtsZs. The model organisms have provided an easy and reliable platform for the study and visualization of the activity of anti-FtsZ inside the cells. Several studies have demonstrated the effect of the drugs on *Sa*FtsZ, but only a few of the studies have explored the effect of the compounds on *Mtb*FtsZ, *Spn*FtsZ, and *Pa*FtsZ (Table 1). While inhibition of GTPase activity and polymerization of *Ec*FtsZ or *Bs*FtsZ provide a reliable indication of the FtsZ-targeting action of the compounds, does the drug show the same effect on FtsZ from other bacteria? Several examples from these studies given below demonstrate that it is not always the case.

We have recently shown that anti-FtsZ agents might show different effects in different bacteria with different phenotypes of FtsZ inhibition. Vitamin K3, a biomolecule of the vitamin K family, was shown to display differential effects on the FtsZ inhibition in *B. subtilis, S. pneumoniae*, and *E. coli* [146]. By immunostaining against FtsZ, it was observed that Z-ring disruption was observed in both the bacteria upon treatment with Vitamin K3; however, in *B. subtilis* there were punctate spots of FtsZ observed in the DNA-free regions of the cells while in *S. pneumoniae* no such spots were seen. Further, Vitamin K3 did not show inhibition of *E. coli* or its FtsZ [146]. This goes to show that Vitamin K3 might have different binding pockets within different FtsZ, thereby showing different effects on them or none at all (as in the case of *E. coli* FtsZ). Another similar compound, plumbagin also showed inhibitory activity against only *Bs*FtsZ while it did not affect *Ec*FtsZ [147]. Several other drugs mentioned in Table 1, also do not affect *Ec*FtsZ, while they show good activity against *Bs*FtsZ.

Evidence of anti-FtsZ targeting agents having differential binding in different FtsZ also comes from the studies on PC190723 [148–151]. PC190723 was shown to be a potent inhibitor of *S. aureus* with an anti-FtsZ mode of action [148]. PC190723 stabilized the polymerization and reduced the GTPase activity of *Sa*FtsZ and *Bs*FtsZ, and did not show any effect on *Ec*FtsZ, thereby showing PC190723 could be a gram-positive bacterial inhibitor [149]. PC190723 was tested against gram-positive *Enterococcus faecalis* and *S. pneumoniae* and their FtsZ [150]. It was observed that these gram-positive bacteria were resistant to PC190723 and their FtsZs were not affected by the drug [150]. Two amino acids are different in the binding pocket of FtsZ from *E. faecalis* and *S. pneumoniae* compared with FtsZ of *B. subtilis* and *S. aureus* which was attributed to the resistance of these bacteria [150]. Additionally, computational studies established that PC190723 had different binding pockets for different FtsZ from bacteria [151].

The differences in binding affinities of a drug towards different FtsZ also sheds a light on the differences in FtsZ binding pockets. An example would be of a guanidinomethyl biaryl compound 13, which shows similar binding affinities between *Sa*FtsZ and *Ec*FtsZ, but shows lower affinity toward FtsZ from *E. faecalis* [152]. Synthetic inhibitors UCM05 and UCM44 inhibited the polymerization of *Bs*FtsZ, but, stabilized and induced the polymerization of FtsZ from *M. jannaschii*, even though the binding pockets were similar [153]. This indicates that the differential activity arose not at the binding stage, but during filament formation. Further, UCM05 and UCM44 did not show a significant effect on *Ec*FtsZ [153].

## Challenges and opportunities

Even though several anti-FtsZ agents have been identified, no drug has been translated to the clinics. Only one FtsZ-targeting agent TXA709, a bioavailable prodrug of TXA707, is currently undergoing clinical trials [154]. The roadblocks to transitioning of an anti-FtsZ agent from pre-clinical studies to the clinical stage include the lack of broad specificity and low *in vivo* efficacy. Only a few FtsZ inhibitors have been identified that target both gram-positive and gram-negative organisms. To address these challenges, there needs to be a closer inspection of the characteristics of FtsZ that can render it ‘druggable’.

### Lessons from the pre-clinical development of an FtsZ-targeting anti-Staphylococcal drug

An FtsZ-targeting compound, PC190723 (3-[(6-chloro[1,3]thiazolo[5,4-b]pyridin-2-yl)methoxy]-2,6-difluorobenzamide) was discovered to be highly potent against methicillin-resistant *Staphylococcus aureus* (MRSA) [148,149]. Even though PC190723 and its derivates with increased bioavailability showed promising *in vivo* efficacy in mouse model, due to poor solubility issues the clinical development of this compound as an anti-staphylococcal drug was hindered [148,155]. A group with TAXIS Pharmaceuticals Ltd. designed the prodrug TXY436 with improved solubility [156] and TXY541 with improved stability, showed potent *in vivo* efficacy [157]. Further derivation resulted in the design of the prodrug TXA709 that was resistant to metabolic attack [158]. Its active drug TXA707 (Figure 7 and Table 1) is a potent bactericidal agent against multidrug-resistant *S. aureus* showing low toxicity to mammalian cells [158]. Efficient *in vivo* translation of the anti-staphylococcal activity demonstrated in murine infection models established TXA709 as a promising candidate for clinical development [159]. TXA709 recently underwent in-human Phase 1 clinical trials and was shown to have no serious adverse events [160]. It is currently being developed as a combinatorial drug with conventional antibiotics for the oral treatment of MRSA [161]. TXA6101 is another derivative that is effective against mutated FtsZ from MRSA that has developed resistance to TXA707 [162]. From the crystal structures of TXA707 and TXA6101 bound to FtsZ [162], it is interesting to note that TXA6101 shows structural flexibility compared to TXA707. This enables it to target the FtsZ from gram-negative bacteria as well, broadening the specificity of these compounds to previously less targeted class of bacteria with respect to anti-FtsZ therapy [163]. TXA6101 and TXA6129 (Figure 7 and Table 1) show activity against *Enterobacteriaceae* family of pathogens when used in combination with efflux pump inhibitors [163]. As seen with several anti-FtsZ agents in section 4.3, these compounds inhibit *E. coli* FtsZ polymerization and bundling, in contrast to PC190723 which stabilizes *S. aureus* FtsZ polymers [149,163].

#### Exploring FtsZ from pathogenic organisms for anti-FtsZ studies

The examples in the previous section, highlight the need to further understand the ‘conserved’ protein FtsZ, which seems to have a species-specific behavior. By considering only model rod-shaped organisms for elucidating the mechanism of action, there is a significant limitation to the type of effects of the drugs that are studied. The pathogens against whom these drugs need to be identified have several unique and dissimilar characteristics compared to model rods. Screening libraries of potential FtsZ inhibitors in pathogenic organisms will aid in the process of identifying successful candidates for pre-clinical studies. Even if the identified candidates do not show broad-spectrum activity, the established activity of the drug against the pathogen will serve as a platform for the direct development of narrow-spectrum antibiotics. With the alarming increase in antibiotic resistance, narrow-spectrum antibiotics will also become essential for urgent treatment [164].

#### Alternative binding pockets in FtsZ

Considering the limited binding pockets of FtsZ that have been explored, an interesting binding region would be the C-terminal tail region. Even though several regions of the CTT, like CTL and CTV, are not conserved, the intrinsically disordered region of CTT seems to be important for the functioning of the protein, and for its interactions with other proteins of the divisome. Inhibiting the flexibility of the CTT can be a potential strategy for inhibiting the binding of several accessory proteins to FtsZ. Given that targeting intrinsically disordered regions is challenging, several computational approaches that have yielded successful drugs for other intrinsically disordered proteins can help [165]. One such example is the targeted covalent inhibitors that have shown promising activity against the intrinsically disordered protein, tau [166]. A very recent study involved the design of several peptides with multiple arginine residues that bind to negatively charged residues of the tubulin C-terminal tail [167]. The binding of these macrocyclic peptides to CTT caused the promotion of tubulin polymerization and the shortening of the nucleation phase [167]. This study introduces a viable strategy for targeting the intrinsically disordered CTT of tubulin, that can inspire the development of new strategies for targeting the CTT of FtsZ. Several proteins are known to bind the CTT region of FtsZ, many of which could share the same binding regions. An important finding in this regard is the same binding pocket on *Corynebacterium glutamicum* FtsZ for two structurally and functionally different proteins SepF and SlmA [110]. This encourages the challenging endeavor of identifying anti-FtsZ agents that target the CTT to impair several cell division-related interactions in the cell.

#### New strategies

With the discovery of the conformational switch of FtsZ during polymerization, arresting the protein in one of the conformational states is an attractive strategy to target FtsZ [99,102,168,169]. With the inhibition of the conformational change, assembly of FtsZ is prevented, rendering the Z-ring non-dynamic. Inhibitors targeting the inter-domain cleft may serve as potential leads for the design of such inhibitors, that need to bind to the C-terminal domain of FtsZ involved in the conformational change.

Whatever may be the inherent differences between the assembly characteristics of FtsZ from different bacteria, the fact remains that FtsZ is a central player in the cell division mechanism that interacts with several different proteins that are regulated spatiotemporally based on the shape and type of bacteria. This expands the target of anti-bacterial action from just FtsZ to those essential FtsZ-associated proteins and their interacting regions. Also, designing inhibitors based on the interactions between FtsZ and its associated proteins is an emerging field of study [170–172]. However, more research must be done for a clearer understanding of the interaction of FtsZ and its associated proteins, structurally and functionally, before inhibitors can be designed based on it.

## Conclusion

FtsZ from different bacteria show different characteristics *in vitro*, bundling type, GTPase activity, and binding to cations [103,173]. Differences in assembly behavior may also arise due to the C-terminal tail region [119]. The differences in structure and functionality of FtsZ extend to the myriad of functions that the cytoskeletal protein performs within the cells. The differences observed in gram-positive and gram-negative bacteria may arise due to the thicker cell wall of the former, requiring different rates for treadmilling of FtsZ and subsequent assembly of peptidoglycan in the septum. FtsZ treadmilling may also be highly influenced by the accessory proteins which have several unique bacteria-specific roles. It is noted that these differences arise even at the species level. For example, key differences between elongation and PG incorporation between *M. tuberculosis* and *Mycobacterium smegmatis* have been recently reported [174]. This further emphasizes that the cell division machinery operates in a species-specific manner. Through this review, we have given a glimpse into the important differences in the cell division machinery and FtsZ in some of the pathogenic species, to reiterate the urgent need for their study. Even though the rods *B. subtilis* and *E. coli* have been the backbone research models for cell division studies, the scientific community is now expanding the horizons to pathogenic organisms. More and more evidence of FtsZ being the central protein that has several previously unidentified functions throughout the cell growth is established every day, emphasizing that FtsZ is an excellent target for anti-bacterial therapy. Several anti-bacterial agents that target FtsZ would be tested and studied in pathogenic organisms. With the era of antimicrobial resistance looming over the world, anti-bacterial therapeutic research has much to gain from these studies.

## Abbreviations

AMR: anti-microbial resistance
CTC: C-terminal conserved
CTL: C-terminal linker
CTT: C-terminal tail
CTV: C-terminal variable
MRSA: methicillin-resistant *Staphylococcus aureus*
PBP: penicillin-binding proteins
PG: peptidoglycan
